# Supplementary material for: Differential impacts of freshwater and marine covariates on wild and hatchery Chinook salmon marine survival
Source: PLoS One. 2021 Feb 9;16(2):e0246659. doi: 10.1371/journal.pone.0246659 (PMC7872236; doi:10.1371/journal.pone.0246659)
Supplement: S1 Table — All files and model output are available for upload from the github.com/bchasco/sar_paper. (DOCX) [file pone.0246659.s001.docx]

S1 Table. **R scripts.** All files and model output are available for upload from the github.com/bchasco/sar_paper.

| File | Description | Output |
| --- | --- | --- |
| create_DataAndPars.r | Creates a list of data and parameter objects that are used by the TMB obj | data (list)  parameters (list) |
| create_MapAndObj.r | Creates a map list of parameters and random effects that are not to be estimated by the TMB object, Obj. | myMap (list)  Obj (TMB) |
| wrapper_modelRuns.r | A wrapper that takes user defined inputs and then run the optimization for the TMB object. | rep (list) – TMB output  SD (list) – TMB output |
| wrapper_simRuns.r | A wrapper to do the simulation testing for the parameters of the best fit model to the wild spring/summer Chinook salmon | simMelt (data.frame) – estimated parameters for the simulated data sets |
| wrapper_simQuadratic.r | A wrapper to compare the parameters estimates for the simulated data based on our AR1 model for day and day/year interaction model with a mixed-effect model where day effect is a fixed effect described linear combination of day and day^2^ | simMelt (data.frame) – estimated parameters for the simulated data sets |
| fig_AnnualSurv_ggplot.r | Plot of annual survival | fig_AnnualSurv_ggplot.tiff |
| fig_DailySurv_ggplot.r | Plot of daily survival, aggregated across years | fig_DailySurv_ggplot.tiff |
| fig_DayXYearSurvival_ggplot.r | Plot of daily survival by year | fig_DayXYearSurvival_ggplot.tiff |
| fig_envEffect_ggplot.r | Plot of the environmental effects | fig_envEffect_ggplot.tiff |
| fig_EnvironmentalVariableWt_ggplot.r | Plot of the predictive ability of the different environmental covariates | fig_EnvironmentalVariableWt_ggplot.tiff |
| table_AIC.r | Table of the AIC values for the top models | table_AICOutput.csv |
| table_bestFitMods.r | Table of best-fit models for hatchery and wild fish | table_bestFitMods.csv |
| table_resDeviance.r | Table of residual deviances for different fixed and mixed-effect models | table_resDeviance.csv |
