# Supplementary material for: Differential impacts of freshwater and marine covariates on wild and hatchery Chinook salmon marine survival
Source: PLoS One. 2021 Feb 9;16(2):e0246659. doi: 10.1371/journal.pone.0246659 (PMC7872236; doi:10.1371/journal.pone.0246659)
Supplement: Attachment — Submitted filename: Response to reviewers.docx [file pone.0246659.s004.docx]

Minor comments:

“carryover effects” – The introduction brings in the idea of carryover effects, but the link between carryover effects and arrival timing in the estuary is weakly developed in the introduction. That is, at first glance, arrival timing itself does not seem to be a carryover effect from freshwater to the estuary/ocean. Size at outmigration, if size is mediated by the freshwater environment, is an example of a direct measure of a carryover effect if size at outmigration subsequently affects SAR. The argument for arrival timing as a carryover effect is better supported in the discussion. However, because carryover effects are such an important concept in this paper, I think it would be worth spending a bit more time in the introduction to explain to the reader why arrival timing can be considered as a carryover effect. If you can get across the idea that the freshwater environment itself (and management thereof) can influence arrival timing, and arrival timing affects SAR, then it better establishes the idea of arrival timing is a carryover effect and will better set up the rest of paper.

**We agree that migration timing should feature more prominently and have included the following addition to the fourth introductory paragraph.**

**Carryover effects are effects that “carry over” from one life stage to another [17]. We propose the working definition: in an ecological context, carryover effects occur in any situation in which an individual’s previous history and experience influences their current performance in a given situation. While effects such as length, weight, and freshwater environmental covariates may be explored in future analyses [7], we focused our attention on how phenology (i.e., migration timing) in the freshwater environment (the previous history) carries over to the survival in the marine environment (the current life stage). In part, we have focused our attention on phenology because of the large emphasis that co-managers of the Columbia Basin have placed on restoring the natural migration conditions for juvenile salmon. In particular, the practice of transporting fish in barges around dams and spilling more water over the top of dams has decreased delay, resulting in fish arriving at the estuary earlier.**

The analysis is conditioned on fish detected at Bonneville Dam. So what are the consequences of ignoring fish that survived to Bonneville but were not detected? One thought is that “annual” survival should be interpreted with caution, as this is the weighted average survival of fish detected at Bonneville. Although unbiased with respect to this strict definition (fish detected at Bonneville), it could be a biased estimate for all PIT-tagged spring/summer Chinook, particularly if detection probability varies with day of year (which is likely). Second, Faulkner et al. (2019; <https://doi.org/10.1002/tafs.10200>) recently found that detection probability at Columbia River dams increased with fish size, suggesting that the analysis by Chasco et al. uses fish that are likely to be larger than the run-at-large passing Bonneville Dam. Conditioning on detected fish vastly simplifies modeling, and although I don’t think this is a major issue, the authors should at least explicitly acknowledge that 1) many PIT-tagged fish passing Bonneville are not included in the analysis, and 2) the potential consequences of such exclusion.

**We agree with the reviewer on this point as well and thank them for directing us to the Faulkner paper. We have added the following text in the discussion.**

**We note that although fish size is known to affect migration pathways through the hydrosystem (Faulkner et al. 2019), detections at Bonneville Dam did not show a significant size bias (see Fig 3 in Faulkner et al 2019). Nonetheless, we acknowledge that there may be other ways in which detected fish were not fully representative of the run-at-large, so our conclusions apply specifically to this set of fish. Further research to extend this model is necessary to fully understand how the interaction of other fish attributes such as size in the freshwater environment are likely to affect marine survival.**

Line 85: “barged downstream as juveniles”. Explain more fully otherwise readers outside of the Columbia Basin will have no idea what this means.

**We have added,**

**Lines 77 – 79, In particular, the practice of transporting fish in barges around dams and spilling more water over the top of dams has decreased delay, resulting in fish arriving at the estuary earlier.**

**and**

**Lines 108 to 110, and fish that did not volitionally migrate (i.e., placed into barges to avoid passage through the hydrosystem) downstream as juveniles**

Lines 85-86: Why exclude fish migrating prior to April 9th and after July 8th? The fact the that they comprise <0.14% of the total observations is just as much an argument for including these observations as excluding them.

**We added the following,**

**There is very little data to inform the temporal autocorrelation at the margins of the migration period, and an initial analysis demonstrated that removing these observations greatly improved the speed and convergence of the model fit with little change in the estimates of model parameters.**
