# Supplementary material for: Differential impacts of freshwater and marine covariates on wild and hatchery Chinook salmon marine survival
Source: PLoS One. 2021 Feb 9;16(2):e0246659. doi: 10.1371/journal.pone.0246659 (PMC7872236; doi:10.1371/journal.pone.0246659)
Supplement: Attachment — Submitted filename: Response2.docx [file pone.0246659.s005.docx]

**Editor’s comments:**

1. I recommend following Edwards and Augre-Mth ([2018](https://besjournals.onlinelibrary.wiley.com/doi/full/10.1111/2041-210X.13105)) guidance on scientific notation. For example, they suggest avoiding labels in superscripts if possible to avoid confusion, as in equation 7 (is D an exponent or a label?), using italics when using English letters are used for variables, using lower case bold for vectors and upper case bold for matrices, etc. Also, I suggest using lower-case sigma^2 to represent variance terms, with labels in the subscript to differentiate variance terms (and upper case Sigma for a covariance matrix, as you’ve done). Also, given that rearing types (hatchery and wild) were modelled independently, you could keep the r subscript out of the equations entirely and just mention that you ran the model on two data sets: wild and hatchery. This would simply the equations significantly.   [Edwards, AM, Auger‐Méthé, M. Some guidance on using mathematical notation in ecology. Methods Ecol Evol. 2019; 10: 92– 99. <https://doi.org/10.1111/2041-210X.13105>]

We have updated the equations in accordance with the format suggested by Edwards and Auger-Methe.

2. How were the environmental variables in Table 2 chosen? Some justification for their choice would be valuable.

We have included the following text on lines 141 to 151

Variables represent large-scale oceanographic patterns as well as regional physical metrics. While not all variables have a proposed direct mechanistic relationship with salmon survival, they have been shown to correlate with Chinook salmon returning to specific ESUs [20,21]. The environmental variables in our models include basin-scale oceanic and atmospheric variables (i.e., North Pacific Gyre Oscillation (PGO), Oceanic Niño Index (ONI), Multivariate ENSO Index (MEI), and North Pacific Index (NPI)), local or regional variables (i.e., sea surface temperature for coastal Washington (ersstWACoast), sea surface temperature from Johnstone and Mantua (2014) (ersstARC), coastal upwelling index (CUI), and measure of Sverdrup index most correlated with the temperatures in the upper 20 meters (transport)), and indicators from the Columbia River representing the environment that salmon inhabited just prior to migrating into the ocean (i.e., Columbia River flow (CRflow) and Columbia River temperature (CRtemp)).

3. Text justifying the inclusion of random effects would also be useful. For example, in the introduction, I suggest a more complete description of why day and year variables were treated as random effects, given this is a key advance over previous studies (as described in the 1^st^ paragraph). I suggest moving the justification for work on page 15 (top of paragraph 2) to the Introduction. Also, why might the day/year interaction term be biologically meaningful, justifying its inclusion? If previous studies did not include autocorrelation in day and year terms, why did you consider this a shortcoming, justifying its inclusion here (is there evidence for autocorrelation in those factors, or autocorrelation in the underlying biological mechanisms for the day and year effects)? This is perhaps especially important given that you found including all random effects resulted in model over-parameterization.

We included the following text in lines 79-101. Some of the text currently in lines 89-105 had previously been included in the discussion – as per the editors comments.

Previous estimates of smolt-to-adult returns (SAR) used either generalized linear models (glm) and treated the temporal variability in survival with fixed effects for day, day^2^, and the day/year interaction [6], or generalized mixed effects models (glmm) with day and or year as independent uncorrelated random effects [7]. Numerous research efforts have shown that not accounting for autocorrelation in fisheries data can lead to biases in parameters estimates and derived variables of the models [19], and it is unknown whether the fixed effects for day and day/year interactions can lead to biases in the expectation and uncertainty in salmon survival models.

In this effort, we provide a generalized statistical model that scientists and managers can use to integrate the complex interacting effects of environmental conditions across multiple habitats with estimates of salmon survival. From a modeling perspective, our justification for including autocorrelated random effects for the day, year, and day/year interactions recognizes that natural systems have inherent variability that a fixed effect model is unlikely to capture, and a random effects model allow us to decouple the uncertainty in the day effect from the uncertainty in the observations. From a biological perspective, other factors not measured during the migration period are also likely to affect salmon survival (e.g., predation) and therefore the effect of migration day may not be as smooth as a quadratic curve that is constant among years. Our model is meant to provide a parametric estimate of the autocorrelation at the daily and annual time scales, recognizing that these data were collected over time and the independence between observations is likely to be affected by the duration between observations. To evaluate our approach relative to previous models of salmon survival, we applied a random effects model to a rich dataset of over 285,000 individually-tagged Snake River spring/summer Chinook salmon between 2000 and 2015.

**Reviewer #2:**

1) The term multivariate seems to be used in the text to refer to a model with multiple predictor variables. Strictly speaking, multivariate refers to a model with more than one response variable. However, the authors only have one response variable in their model (SAR), so the term multivariate should not be used.

We have removed multivariate from the paper.

2) Page 9, equation 2 and associated text. If beta is a vector, please present in in bold. The term x_t should be matrix (where rows are measurements and columns are the different predictors), not a vector as described in the text. The text should be corrected and the term should be represented as a bold capital X. Correction should be applied to Table 3 as well.

We have corrected the equations throughout as per the suggestions of the reviewer and the editor

3) Page 11, first paragraph. The text implies that SAR (s_rjt) is binomially distributed. Actually, s_rjt is a parameter of the binomial distribution. What is binomially distributed is the number of survivors (k_rjt). Please revise.

The sentence now reads,

Given the total number of juveniles that migrated downstream $n_{jt}$ and the predicted SAR $s_{\mathrm{jt}}$, the number of juvenile fish $k_{jt}$ that survived to adulthood and were detected at one of the eight main-stem on the Columbia River and Snake River is binomially distributed

4) The authors frequently refer to the top model(s) as the models that had best-fit. AIC is not simply a measure of fit but rather it is a statistics that represents a balance between model fit and complexity. Therefore, it's not appropriate to refer to the selected model(s) as the model(s) of best-fit (a model with best fit may not even be included among the selected models). I suggest referring to selected models as more parsimonious or optimal model(s).

We have removed best-fit throughout and changed the text to use the term parsimonious.

5) Page 16. The authors refer to the intervals as credible intervals. The term credible is used for interval determined using Bayesian inference, but it seems the authors are using a frequentist inference framework. So, to avoid confusion I suggest they refer to the interval as confidence interval throughout the paper.

We have changed the text to confidence interval throughout.

6) To my knowledge, marginal AIC should not be used to conduct model selection in linear mixed models in the way it was conducted by the authors (i.e. comparing models with different fixed and random effects structures). I suggest the authors check out the paper by Vaida & Blanchard (2005) and the book by Zuur et al. (2009). In the latter, the authors suggest to first select the optimal random effects structure by applying AIC to models with all fixed effects but different random effects. Once the optimal random structure is selected, AIC can then be used to compare models that have different fixed effects but the same (previously selected) optimal random effects structure.

We have considered reviewer 2’s #6 comments carefully. We see their argument as containing to two parts.

1. Vaida is one of many papers about conditional AIC, which should minimize predictive variance for data given predicted random effects. The marginal AIC that we use minimizes predictive variance for new random effects.
2. Zuur suggests selecting random effects given specified fixed-effect structure when using REML.  We are not using a restricted maximum likelihood, so again we feel that mAIC is appropriate.

Additionally, we recognize that this was an important consideration for the editor and the reviewer, so we included the following text in the discussion where we addressed model fit. We are happy to include this explanation in the paper:

We recognize that there are multiple ways to evaluate model fit and specification (i.e., fixed and random effects structures) for mixed effects models. For instance, Vaida and Blanchard [46] propose using conditional AIC, because marginal AIC tends to favor smaller models with fewer random effects [47]; however, conditional AIC is not computationally reasonable for large datasets such as ours [47]. Zuur [48] proposed, in the case of REML models (restricted maximum likelihood), selecting the number random effects using marginal AIC conditioned on all of the fixed effects, and then choosing the fixed effects conditional on the structure of the optimal random effects. Because we have not implemented REML in our joint likelihood, and given that we have greater than 1600 observations [47], we have chosen to compare models using the marginal AIC and recognize that we may be underestimating the optimal number of random effects. Future analyses may also consider using conditional AIC approaches (e.g., the DHARMa package in R [49]) to evaluate model misspecification for a larger suite of random effects models; however, in this paper we have specifically focused on evaluating the effects of the unknown processes for day and year.

7) It seems Figs. 4 and 5 were uploaded in the wrong order. Please check.

We checked and they were uploaded in the correct order.

Brandon Chasco and others
