# Supplementary material for: Differential impacts of freshwater and marine covariates on wild and hatchery Chinook salmon marine survival
Source: PLoS One. 2021 Feb 9;16(2):e0246659. doi: 10.1371/journal.pone.0246659 (PMC7872236; doi:10.1371/journal.pone.0246659)
Supplement: Attachment — Submitted filename: Response to reviewers.docx [file pone.0246659.s006.docx]

PONE-D-20-13616R1
Differential impacts of freshwater and marine covariates on wild and hatchery Chinook salmon marine survival
PLOS ONE

Below are a few minor revisions that I invite you to consider:

Line 91. I suggest adding the words  “structure in “ after inherent. By including autocorrelation and random effects, the model is providing some structure to the variability, e.g.,  deviations this year is related to deviations last year, and year and day effects on SAR are drawn from common random distributions (Eqns. 4 and 5, respectively).

We have added the suggested words to the text.

Line 194, Suggest revising to “Where the elements of matrix **Σ**, ...” for clarity.

Eqn. 7. This equation makes more sense to me in the original form: sig^2/(1-rho^2) x rho^delta, which is more consistent with equations 4 and 5. Although they are equivalent, the original form might be more intuitive to readers. (Same for the equation on line 195).

We have added the following text that combines the comments related to the text and equation 7,

$\Sigma_{j,j+\delta}=\sigma^{2}\frac{\rho^{\delta}}{1-\rho^{2}}$	(7)

Where the elements of the covariance matrix $\left( \boldsymbol{\Sigma} \right)$ are a function of the variance parameter $\sigma^{2}$ which is rescaled based on the correlation $\rho$ between days and the $\delta$ number of days between observations.

Table 5, Suggest adding subscripts 1 and 2 (or PDO.sum and CUI.spr as in Fig. 6) to the beta terms (the effects of first and second marine covariates, respectively). I assume these are elements of the vector **β**. Also, in this table, I suggest aligning the Parameter description with the Symbol. It looks like they are currently offset by 1 line.

| Effect of first marine covariate^1^ | $\beta_{1}$ | 0.488 ( 0.228, 0.747 )^2^ | 0.478 ( 0.218, 0.737 )^2^ | 0.458 ( 0.22, 0.695 )^3^ |
| --- | --- | --- | --- | --- |
| Effect of second marine covariate^1^ | $\beta_{2}$ | 0.547 ( 0.283, 0.812 )^2^ | 0.56 ( 0.295, 0.825 )^2^ | -0.608 ( -0.82, -0.396 )^3^ |

**^1^**The first and second marine covariates are elements of the covariate vector $\boldsymbol{\beta}$.

**^2^**For hatchery spring/summer Chinook salmon, the first and second marine covariates in the model with the lowest AIC were transport.sum and npgo.sum, respectively.

**^3^**For wild spring/summer Chinook salmon, the first and second marine covariates in the model with the lowest AIC were cui.spr and pdo.sum, respectively.

Yes, I recommend including the suggested paragraph on conditional vs marginal AIC.
